# Methods for objectively assessing clinical masticatory performance: protocol for a systematic review

**DOI:** 10.1186/s13643-016-0403-5

**Published:** 2017-01-26

**Authors:** Per Elgestad Stjernfeldt, Inger Wårdh, Mats Trulsson, Gerd Faxén Irving, Anne-Marie Boström

**Affiliations:** 1 Dental Clinic of Medical Dental Care at Stockholms Sjukhem, Mariebergsgatan 22B, 112 19 Stockholm, Sweden; 2 Akademiskt Centrum för Äldretandvård, Academic Center for Geriatric Dentistry, Stockholm, Sweden; 3 Department of Dental Medicine, Division of Gerodontics, Karolinska Institute, Box 4064, 14104 Huddinge, Sweden; 4 Department of Dental Medicine, Karolinska Institute, Box 4064, 14104 Huddinge, Sweden; 5 Department of Neurobiology, Care Science and Society, Division of Clinical Geriatrics, Karolinska Institute, Blickagången 6/Hälsovägen 7, 14157 Huddinge, Sweden; 6 Department of Neurobiology, Care Science and Society, Division of Nursing, Karolinska Institute, Box 23300, 141 83 Huddinge, Sweden; 7 Department of Geriatric Medicine, Danderyd Hospital, SLSO, 182 87 Danderyd, Sweden; 8 Department of Nursing, Western Norway University of Applied Sciences, Haugesund, Norway

**Keywords:** Masticatory performance, Study protocol, Reliability, Validity, Compromised dentition, Older adults

## Abstract

**Background:**

Chewing and masticatory function constitutes one of the most important oral health factors that affect quality of life, especially in older individuals. Little consensus currently exists regarding ways to objectively assess clinical masticatory performance (in this context, *performance* refers an individual’s objective ability to mix or comminute food bolus). That said, many methods were developed to assess masticatory performance. Consequently, systematic review of the literature would be of great value when it comes to identifying various methods for objectively assessing clinical masticatory performance and for evaluating these methods.

**Design:**

This study protocol describes a systematic review that intends to (i) identify methods to objectively assess clinical masticatory performance and (ii) evaluate psychometric properties (such as validity and reliability) of the identified methods. A systematic literature search is required to do so in these sources: MEDLINE (Ovid), Embase (embase.com), Web of Science Core Collection (Thomson Reuters), Cochrane (Wiley), and Cinahl (Ebsco).

Inclusion criteria: studies in scientific, full-text articles; development articles; validation articles; studies of the general adult population, ages ≥18. Exclusion criteria: topics and article types that cover interview methods and self-reported questionnaires; methods/instruments that measure subjective masticatory performance; qualitative studies and case studies; opinion and editorial pieces; animal studies; studies of humans with severe oral health complications.

**Discussion:**

This systematic review will result in a comprehensive assessment of various methods designed to objectively measure clinical masticatory performance. This systematic review will rate these methods, assess their reliability and validity, and identify one or more methods that can be recommended for use in clinical and scientific environments. From what is currently known, no systematic evaluation of various methods for objectively assessing clinical masticatory performance has been published.

**Systematic review registration:**

PROSPERO CRD42016037700

**Electronic supplementary material:**

The online version of this article (doi:10.1186/s13643-016-0403-5) contains supplementary material, which is available to authorized users.

## Background

An increasing number of older individuals retain dentition throughout life. Many have gone through oral rehabilitation with various dental constructions—often with the purpose of securing relevant chewing and masticatory function that has been shown to be one of the most important oral health factors associated with the quality of life in older individuals [1–3].

Many clinical assessment methods were developed to assess masticatory performance, for example, color-changing chewing gums, sieving comminuted food or artificial food, optical scanning of masticated particles, or measuring release of dye when chewing food [4]. Currently, there are many different methods used to clinically assess masticatory performance objectively (here, *performance* refers to someone’s objective ability to mix or comminute food bolus) [4]. For practitioners and researchers, it would be interesting to find out if these methods could be used to evaluate masticatory ability—before dental treatment. For research studies that develop and test interventions—to enhance masticatory performance—need exists for reliable, valid methods that objectively evaluate clinical masticatory performance.

The most common approach to evaluating masticatory performance has been to sieve comminuted food and determine the degree of food breakdown [4–9]. Food or artificial food is chewed with a predetermined number of chewing cycles. Food particles are then sieved, and particle size is analyzed. These comminution tests differ somewhat, although the general methodology is similar. For example, one study indicated that a multiple sieve method was more reliable in determining masticatory performance than a single sieve method [9]. Edlund et al. developed a masticatory efficiency index that could be used in these types of comminution tests, but it also required five separate test sessions to be reliable [10]. Optical scanning of chewed food particles was used in comminution studies—in lieu of sieving [11]. Comminution tests were used to evaluate masticatory performance after implant treatments or to assess masticatory performance in patients with conventional dentures [12, 13].

Another common masticatory performance assessment method involves mixing ability tests. One study indicated that a mixing ability test discriminates better (between groups of individuals with compromised masticatory performance) than comminution tests [14]. In another study, two colored chewing gums were used as test food. Determining the degree of mixing of the two colors assessed mixing ability. Optical/scanning methods or visual inspection enable the assessment [12]. Multicolored paraffin wax, which is mixed during chewing, can be used as test food for determining masticatory performance; this method demonstrated validity and reliability in individuals with normal and compromised dentition [15, 16].

While comminution and mixing tests are common methods when quantifying masticatory performance, others were also developed. Some studies used color-changing chewing gums [17, 18]; here, color change was assessed with a colorimeter [17] or spectrophotometer [18] and was used as a way of quantifying masticatory performance.

While most of these tests were used in laboratory studies, no established method for evaluating a patient’s objective masticatory performance is available within clinical practice, and the clinician must rely on masticatory performance assessments via oral status appraisals, existing dentition, or patients’ subjective experiences. The Eichner index, which is used to classify occluding contacts, was validated as a method in relation to masticatory performance [19].

The term *masticatory function* was used for objective and subjective measurements [4]. In this study protocol, we use *masticatory performance*, because this term relates more to the objective aspects of mastication [4], and it has been adopted in clinical studies [20–22].

Our scoping reviews identified two reviews that were published in this field. Oliveira et al. presented a review of various methods used for evaluation of masticatory performance in patients with conventional complete dentures [23]. Fifty-two articles were analyzed, and Oliveira et al. concluded that despite the wide variety of methodologies, sieve methods are considered to be the golden standard for evaluating complete dentures wearers’ masticatory ability. A second systematic review synthesized available knowledge of distal-extension removable dental prosthesis in individuals with shortened dental arches and its effects on masticatory performance [24]. Four studies provided data on comminution, three on mixing ability, and one on both tests. The authors concluded that distal-extension removable dental prostheses—in subjects with shortened dental arches—partially compensate for reduced masticatory performance. But note that these reviews did not evaluate methodological quality of the studies of various clinical assessment methods used to determine masticatory function. Our scoping reviews of the literature showed that no systematic evaluation of varying methods used to assess objective clinical masticatory performance was published. So it would be of great value to conduct at systematic review that leads to identifying various methods of measuring objective masticatory performance and evaluating psychometric properties of identified methods so that practitioners can:

- Monitor masticatory performance objectively before treatment
- Assess whether treatment is indicated
- Restore masticatory performance
- Evaluate objective masticatory performance after treatment

## Objectives

Primary objectives are to:

– Identify methods for objectively assessing clinical masticatory performance
– Evaluate psychometric properties (such as validity and reliability) of the identified methods

Secondary objectives are to:

– Compare measurement properties of the identified methods
– Identify any types of reported adverse events in development or validation of methods in the selected studies

## Methods

### Protocol/registration

The Preferred Reporting Items of Systematic Reviews and Meta-Analyses Protocol (PRISMA-P) guidelines are to be used for documenting review results [25]. Additional file 1 displays the PRISMA-P checklist. The protocol was registered in the PROSPERO database (Ref: CRD42016037700).

#### Inclusion criteria

Studies will be included in this systematic review if they are reported in scientific, full-text articles. Study designs must describe development of methods that objectively assess clinical masticatory performance in adult individuals (development article) or evaluate measurement properties of methods that objectively assess clinical masticatory performance in adult individuals (validation article).

There will be no restrictions regarding type of:

- Time frame for completing assessments such as before, during, or after dental procedures
- Settings in which assessments were conducted

This systematic review will include studies of the general adult population, ages ≥18

#### Exclusion criteria

These topics and article types will be excluded from this systematic review: interview methods and self-reported questionnaires; methods/instruments that subjectively measure masticatory performance; qualitative studies and case studies; opinion and editorial pieces; animal studies; and studies of humans with severe oral health complications, for example, oral cancer, malocclusions, and trauma (these patient groups represent special dental conditions, problems, and treatments).

If a study is unavailable in full-text, then the abstract will be excluded.

#### Methods for objectively assessing clinical masticatory performance

In this systematic review, clinical masticatory performance is considered a method that assesses an individual’s ability to mix or comminute food/artificial food bolus.

#### Outcomes

Outcomes from this systematic review will be lists/tables/descriptions of:

- Methods for objectively assessing clinical masticatory performance (i.e., articles that report development or validation studies on these types of methods)
- Measurement properties described in the identified methods (i.e., the articles must have reported measurement properties for the method that was developed and validated)
- Adverse events that occurred during method development or validation

#### Language

Scientific articles published in English only will be selected for this systematic review. A list of possible relevant titles in other languages will be provided in an appendix.

### Information sources

These databases will be searched from their inception up to January 2017: MEDLINE (Ovid), Embase (embase.com), Web of Science Core Collection (Thomson Reuters), Cochrane (Wiley), and Cinahl (Ebsco). To complete our searches in the databases, the Google Scholar search engine will be used to search potential databases such as university databases and conference proceedings, among others, for articles. If only abstracts are identified, then authors will be contacted to obtain full-text articles.

The reference lists of included articles or relevant reviews identified in the search will be manually screened. Experts and investigators in the field will be contacted to obtain information on ongoing or unidentified studies.

### Search strategy

The overall search strategy was developed with librarians at Karolinska Institute’s university library. The staff at this library has extensive expertise and experience in developing search strategies for systematic reviews. Library staff will also conduct the literature search.

Search strategies will be developed for each database, with appropriate terms from controlled vocabulary (e.g., MeSH in MEDLINE and Emtree in Embase). Main terms will be mastication, bite force, chewing gum, and deglutition—combined with different terms for measurement properties. Along with the terms from MeSH and Emtree, a broad range of current free-text terms will be used. The free-text terms will, if appropriate, be truncated and/or combined with proximity operators.

A further search will be done with the names of the methods found in the original search. These names will be combined with AND—with the requirements for the target population and measurement properties.

Additional file 2 shows the search strategy in more detail.

### Data management

All citations will be imported into the EndNote (version X7.5) reference program. The library staff will save the electronic searches, and summaries of the searches will be printed to capture results of the searches for the protocol records.

### Study selection

The EndNote reference manager software will be used during the study selection. After the initial search, obvious duplicates will be removed using the reference manager software. One reviewer will perform this procedure. Two independent reviewers will assess remaining titles and abstracts eligibility. If a title or abstract is not enough to determine eligibility, then the full-text article will be obtained.

Full-text articles will be obtained from the remaining eligible abstracts. Two reviewers will independently judge each article for eligibility. If the information in the full-text article is not enough to determine eligibility, then authors will be contacted to obtain missing information. Discussions between the two reviewers will resolve any disagreement. If agreement is not reached, then a third reviewer will decide whether the article will be included. The reasons for excluding an article will be documented. Two team members will independently screen the references lists of the all included articles for any additional relevant studies.

All review team members will receive training in using EndNote and the data extraction forms before procedures in each phase (e.g., screening, eligibility, inclusion, and data extraction) during the systematic review. Training sessions will be organized for calibrating assessments among members. Regular team meetings will be held to discuss issues—to adhere to criteria in each phase. Several abstracts and articles will be pilot tested to ensure agreement and clarify decisions among reviewers.

### Data collection

Two reviewers will implement data extraction for the included articles by using standardized data extraction forms developed for this study (one for development studies and one for validation studies; see Additional file 3). If consensus is not reached, a third reviewer will decide the outcome.

### Data items

Data from included articles will be summarized in standardized data extraction forms that will contain this information: First author, year of publication, title, journal (or type of publication), country of study, study purpose(s), study design, study sample, description of method to objectively assess clinical masticatory performance, and adverse events.

### Outcomes and prioritization (measurement properties of identified methods)

The predefined quality criteria for rating the measurement properties of instruments and methods recommended by the *consensus-based standards for the selection of the health measurement instruments* (COSMIN) group will be used to assess the measurement properties of the different methods [26]. The predefined criteria are in Additional file 4 [26].

The criteria relate to these measurement properties and aspects of measurement properties: reliability (internal consistency, measurement errors, reliability), validity (content validity, structural validity, hypothesis testing), and responsiveness. Also, under consideration will be whether development of any methods included in the systematic review was based on an a priori conceptual framework/model. Two independent reviewers will assess the measurement properties for each method. If consensus is not reached, a third reviewer will decide the outcome.

### Quality assessment of individual studies

The *COSMIN Checklist* will be used to evaluate the methodological quality of the included studies [26]. This assessment is done to avoid including development or validation articles of methods that are of low methodological quality.

Four domains are distinguished in the *COSMIN Checklist*: ratings of the rigor of the reliability, validity, responsiveness, and interpretability with related measurement properties and aspects of measurement properties. Additional file 5 lists these properties. For each of the measurement properties, the *COSMIN Checklist* consists of 5 to 18 items that cover methodological standards. In addition, each item can be scored on a four-point scale (i.e., poor, fair, good, excellent). Taking the lowest rating for each item in one box, an overall score is obtained for each measurement properties separately [26].

### Data synthesis

A final report will be developed; it will summarize the identified methods to facilitate objective assessment of masticatory performance. A table of characteristics for each method will be included. Analysis of the findings will be a process of narrative synthesis to summarize evidence on the measurement properties of the identified methods. First, the methodological quality of the studies will be assessed using the *COSMIN Checklist*. This process will generate a separate rating for each of the measurement properties, where estimated in each study. Second, the estimated measurement properties of the methods will be assessed against the established criteria of measurement properties (see Additional file 4).

### Meta-biases

Meta-bias refers to the biased selection of research findings and covers reporting bias and publication bias. Reporting bias, such as not reporting findings as stated in previous published study protocol, is mainly a concern in articles that report RCT studies and other trials. In development and validation studies, there is no tradition of publishing study protocols before starting data collection in the research study. So this systematic review cannot search for study protocols. The first author will be contacted for articles in which we lack important data or information for conducting our systematic review.

### Confidence in cumulative estimate

The scoring system for the *COSMIN Checklist* will be used to summarize the confidence in cumulative estimate in this review [26]. This scoring system is similar to the GRADE system that is used for synthesizing evidence from clinical trials [27]. The resulting level of evidence for the measurement properties of each method will be classified as per criteria in Additional file 6.

### Differences between the protocol and the review

If criteria or other methods must be changed during review implementation, then all differences will be reported between published protocol and the review. The rationale for each type of change of methods will be documented.

## Discussion

The objective of this systematic review is to conduct a comprehensive assessment of different methods designed to objectively assess clinical masticatory performance. This systematic review will rate these methods, assess their measurement properties, and identify one or more methods that can be recommended for use in clinical or scientific environments. From what is known to us, no systematic evaluation of different methods used to objectively assess clinical masticatory performance has been published.

Poor oral status affects nutritional status and quality of life and leads to impaired function [28, 29]. If the treatment goal of dental practitioner is to restore oral function, then surely masticatory performance would be one of those aspects to consider. This would be of value for all individuals with compromised dentitions—especially older individuals. Recent research has pointed to the relation between cognitive and chewing functions [30]—a factor that makes it very important to find valid, reliable methods to objectively assess clinical masticatory performance.

The strength in this systematic review will be use of the *COSMIN Checklist* to assess measurement properties of the identified methods and methodological quality of included studies [26]. The *COSMIN Checklist* was developed for health status questionnaires. But this checklist has been used in several systematic reviews for evaluating methods such as performance-based measures to assess physical function in hip and knee osteoarthritis [31] multicomponent tools to assess frailty in older adults [32] and functional assessments of older adults at risk of activity and participation limitations [33]. Availability of the university library of Karolinska Institute and its qualified staff is another strength.

One limitation for this review is that it will include articles published in English only. Possible relevant titles for identified articles in other languages will be provided in a list. Identification of methods published in other languages is not expected, and financial resources are unavailable for translation of potential articles. If a seemingly important article in a language other than English is found, then the author(s) will be contacted and a version or information in English will be requested.

Two of the authors have earlier experience in reading and searching for eligible articles for systematic reviews, and all authors will be trained in this work—with the library staff—before the systematic review starts.

## Additional files

Additional file 1: The PRISMA-P checklist. (DOCX 34 kb)
Additional file 2: Search strategy for databases in MEDLINE (Ovid), Embase (embase.com), Web of Science Core Collection, Cochrane (Wiley), and Cinahl (Ebsco). (PDF 97 kb)
Additional file 3: Data extraction form. (DOCX 17 kb)
Additional file 4: Definitions of measurement properties. (DOCX 16 kb)
Additional file 5: COSMIN checklist domains. (DOCX 14 kb)
Additional file 6: Levels of evidence for the quality of the measurement property. (DOCX 13 kb)

## Abbreviations

ACT: Akademiskt centrum för äldretandvård, Academic Center for Geriatric Dentistry
COSMIN: Consensus-based standards for the selection of the health measurement instruments

## References

[1] Strömberg E, Holmèn A, Hagman-Gustafsson ML, Gabre P, Wårdh I (2013). Oral health-related quality-of-life in homebound elderly dependent on moderate and substantial supportive care for daily living. Acta Odontol Scand.

[2] Locker D, Matear D, Stephens M, Jokovic A (2002). Oral health-related quality of life of a population of medically compromised elderly people. Community Dent Health.

[3] Guarnizo-Herreño CC, Tsakos G, Sheiham A, Watt RG (2013). Oral health and welfare state regimes: a cross-national analysis of European countries. Eur J Oral Sci.

[4] van der Bilt A (2011). Assessment of mastication with implications for oral rehabilitation: a review. J Oral Rehabil.

[5] Lucas PW, Luke DA (1983). Methods for analyzing the breakdown of food in human mastication. Arch Oral Biol.

[6] Olthoff LW, van der Bilt A, Bosman F, Kleizen HH (1984). Distribution of particle sizes in food comminuted by human mastication. Arch Oral Biol.

[7] Slagter AP, Bosman F, van der Bilt A (1993). Comminution of two artificial test food by dentate and endentulous subjects. J Oral Rehabil.

[8] Shi CS, Ouyang G, Guo TW (1990). Comparison of food particle distribution masticated by subjects wearing complete dentures and with natural teeth. J Oral Rehabil.

[9] van der Bilt A, Fontijn-Tekamp FA (2004). Comparison of single and multiple sieve methods for the determination of masticatory performance. Arch Oral Biol.

[10] Edlund J, Lamm CJ (1980). Masticatory efficiency. J Oral Rehabil.

[11] van der Bilt A, van der Glas HW, Mowlana F, Heath MR (1993). A comparison between sieving and optical scanning for the determination of particle size distributions obtained by mastication in man. Arch Oral Biol.

[12] Geertman ME, Slagter AP, van Waas MAJ, Kalk W (1994). Comminution of food with mandibula rimplant-retained overdentures. J Dent Res.

[13] Fontijn-Tekamp FA, Slagter AP, van der Bilt A, van’t Hofm MA, Witter DJ, Kalk W (2000). Biting and chewing in overdentures, full dentures, and natural dentitions. J Dent Res.

[14] Speksnijder CM, Abbink JH, van der Glas HW, Jansen NG, van de Bilt A (2009). Mixing ability test compared with a comminution test in persons with normal and compromised masticatory performance. Eur J Oral Sci.

[15] Sato H, Fueki K, Sueda S, Sato S, Shiozaki T, Kato M (2003). A new and simple method for evaluating masticatory function using newly developed artificial test food. J Oral Rehabil.

[16] Sato S, Fueki K, Sato H, Sueda S, Shiozaki T, Kato M (2003). Validity and reliability of a newly developed method for evaluating masticatory function using discriminant analysis. J Oral Rehabil.

[17] Hama Y, Kanazawa M, Minakuchi S, Uchida T, Sasaki Y (2014). Reliability and validity of a quantitative color scale to evaluate masticatory performance using color-changeable chewing gum. J Med Dent Sci.

[18] Hayakawa I, Watanabe I, Hirano S, Nagao M, Seki T (1998). A simple method for evaluation masticatory performance using a color-changeable gum. Int J Prosthodont.

[19] Ikebe K, Matsuda K, Murai S, Maeda Y, Nokubi T (2010). Validation of the Eichner index in relation to occlusal force and masticatory performance. Int J Prosthodont.

[20] Tarkowska A, Katzer L, Ahlers MO. Assessment of masticatory performance by means of a color-changeable chewing gum. J Prosthodont Res. 2016. doi:10.1016/j.jpor.2016.04.004. [Epub ahead of print]. 10.1016/j.jpor.2016.04.004 27211494

[21] Trein MP, Mundstock KS, Maciel L, Rachor J, Gameiro GH (2013). Pain, masticatory performance and swallowing threshold in orthodontic patients. Dental Press J Orthod.

[22] Gameiro GH, Schultz C, Trein MP, Mundstock KS, Weidlich P, Goularte JF (2015). Association among pain, masticatory performance, and proinflammatory cytokines in crevicular fluid during orthodontic treatment. Am J Orthod Dentofacial Orthop.

[23] Oliveira NM, Shaddox LM, Toda C, Paleari AG, Pero AC, Compagnoni MA (2014). Methods for evaluation of masticatory efficiency in conventional complete denture wearers: a systematized review. Oral Health Dent Manag.

[24] Liang S, Zhang Q, Witter DJ, Wanga Y, Creugers NHJ (2015). Effects of removable dental prostheses onmasticatory performance of subjects with shortened dental arches: a systematic review. J Dent.

[25] Moher D, Shamseer L, Clarke M, Ghersi D, Liberati A, Petticrew M, Shekelle P, Stewart LA, PRISMA-P Group (2015). Preferred reporting items for systematic review and meta-analysis protocols (PRISMA-P) 2015 statement. Syst Rev.

[26] Mokkink LB, Terwee CB, Patrick DL, Alonso J, Stratford PW, Knol DL, Bouter LM, de Vet HCW (2010). The COSMIN checklist for assessing the methodological quality of studies on measurement properties of health status measurement instruments: an international Delphi study. Qual Life Res.

[27] Schünemann HJ, Oxman AD, Brozek J, Glasziou P, Bossuyt P, Chang S, Muti P, Jaeschke R, Guyatt GH (2008). GRADE: assessing the quality of evidence for diagnostic recommendations. Evid Based Med.

[28] Smith PA, Entwisthle VA, Nuttall N (2005). Patients’ experiences with partial dentures: a qualitative study. Gerodontology.

[29] Trulsson U, Engstrand P, Berggren U, Nannmark U, Brånemark P-I (2002). Edentulousness and oral rehabilitation: experiences from the patients’ perspective. Eur J Oral Sci.

[30] Lexomboon D, Trulsson M, Wårdh I, Parker MG (2012). Chewing ability and tooth loss: association with cognitive impairment in an elderly population study. J Am Geriatr Soc.

[31] Dobson F, Hinman RS, Hall M, Terwee CB, Roos EM, Bennell KL (2012). Measurement properties of performance-based measures to assess physical function in hip and knee osteoarthritis: a systematic review. Osteoarthritis Cartilage.

[32] Sutton JL, Gould RL, Daley S, Coulson MC, Ward EV, Butler AM, Nunn SP, Howard RJ (2016). Psychometric properties of multicomponent tools designed to assess frailty in older adults: a systematic review. BMC Geriatr.

[33] Wales K, Clemson L, Lannin N, Cameron I (2016). Functional assessments used by occupational therapists with older adults at risk of activity and participation limitations: a systematic review. PLoS One.

