# Supplementary material for: Methods for objectively assessing clinical masticatory performance: protocol for a systematic review
Source: Syst Rev. 2017 Jan 26;6:20. doi: 10.1186/s13643-016-0403-5 (PMC5267407; doi:10.1186/s13643-016-0403-5)
Supplement: Additional file 2: — Search strategy for databases in MEDLINE (Ovid), Embase (embase.com), Web of Science Core Collection, Cochrane (Wiley), and Cinahl (Ebsco). (PDF 97 kb) [file 13643_2016_403_MOESM2_ESM.pdf]

1. Medline (Ovid)

1. exp Mastication/
2. exp Bite Force/
3. (masticat* or oral function or chewed or chewing or bite force or dentition or denture).tw.
4. or/1-3

5. exp Chewing Gum/
6. exp Deglutition/
7. exp Food/
8. exp Dental Stress Analysis/
9. (sieving or sieve or comminution or swallow* or saliva or gum or jell* or wax or food).tw.
10. or/5-9

11. Trail Making Test/
12. Psychological tests/
13. Neuropsychological Tests/
14. Health status indicators/
15. Severity of illness index/
16. Karnofsky performance status/
17. Sickness impact profile/
18. Psychometrics/
19. "Reproducibility of Results"/
20. (measur* or assess* or evaluat* or test* or inventor* or battery or indicator* or profile or index or indices or scale* or instrument* or psychometric* or reproducibility or reliable or reliability or valid*).tw.
21. or/11-20

22. 4 and 10 and 21

2. Embase (embase.com)

No.	Query
#18	**#9** OR **#10** OR **#11** OR **#12** OR **#13** OR **#14** OR **#15** OR **#16** OR **#17**
	**measur***:ab,ti OR **assess***:ab,ti OR **evaluat***:ab,ti OR **test***:ab,ti OR **inventor***:ab,ti OR **battery**:ab,ti OR **indicator***:ab,ti OR **profile**:ab,ti OR**index**:ab,ti OR **indices**:ab,ti OR **scale***:ab,ti OR **instrument***:ab,ti OR **psychometric***:ab,ti OR **reproducibility**:ab,ti OR **reliable**:ab,ti OR**reliability**:ab,ti OR **valid***:ab,ti
#16	**'reproducibility'**/de
#15	**'psychometry'**/de
#14	**'sickness impact profile'**/de
	**'karnofsky performance status'**/de
#12	**'severity of illness index'**/de
#11	**'health status indicator'**/de
#10	**'neuropsychological test'**/de
#9	**'psychologic test'**/de
#8	**#4** OR **#5** OR **#6** OR **#7**
#7	**sieving**:ab,ti OR **sieve**:ab,ti OR **comminution**:ab,ti OR **swallow***:ab,ti OR **saliva**:ab,ti OR **gum**:ab,ti OR **jell***:ab,ti OR **wax**:ab,ti OR **food**:ab,ti
#6	**'food'**/exp
#5	**'swallowing'**/exp
#4	**'chewing gum'**/exp
#3	**#1** OR **#2**
#2	**masticat***:ab,ti OR **'oral function'**:ab,ti OR **chewed**:ab,ti OR **chewing**:ab,ti OR **'bite force'**:ab,ti OR **dentition**:ab,ti OR **denture**:ab,ti
#1	**'mastication'**/exp

3. Web of Science Core Collection

**TOPIC:** (masticat* OR “oral function” OR chewed OR chewing OR “bite force” OR dentition OR denture)<br><br>*AND*<br><br>**TOPIC:** (sieving OR sieve OR comminution OR swallowing OR swallow OR saliva OR gum OR “gummy jelly” OR “gummy jellies” OR jelly OR wax OR food OR “chewing gum” OR deglutition)<br><br>*AND*<br><br>**TOPIC:** (measure* OR evaluat* OR assess* OR test* OR index OR indices OR scale* OR performance OR inventor* OR battery OR indicator* OR profile OR instrument* OR psychometric* OR reproducibility OR reliable OR “reliability or valid*” OR “Trail Making Test” OR “Psychological test*” OR “Neuropsychological Test*” OR “Health status indicator*” OR “Severity of illness index” OR “Karnofsky performance status” OR “Sickness impact profile” OR Psychometric* OR "Reproducibility of Results")

4. Cochrane (Wiley)

Abstract, title, keywords: (masticat* OR “oral function” OR chewed OR “bite force” OR chewing OR “mastication force” OR “masticatory apparatus” OR “masticatory force” OR dentition OR denture)

AND

Abstract, title, keywords: (sieving OR sieve OR comminution OR swallowing OR swallow OR saliva OR gum OR “gummy jelly” OR “gummy jellies” OR jelly OR wax OR food)

AND

Abstract, title, keywords: (index OR performance OR cognition OR efficiency OR scores OR score OR scoring OR assay OR questionnaire* OR question* OR study OR studies OR sample OR sampling OR index OR impairment* OR mixing OR intervention OR subject* OR software OR computer analysis OR “computer method” OR ability OR determinant* OR determine OR participant* OR measure* OR assess* OR evaluat* OR estimat* OR test*)

5. Cinahl (Ebsco)

1. (MH "Mastication") OR
2. (MH "Bite Force")
3. TX (masticat* or oral function or chewed or chewing or "bite force" or dentition or denture)
4. or/1-3

5. (MH "Chewing Gum")
6. (MH "Deglutition") OR (MH "Deglutition Disorders")
7. (MH "Food+")
8. TX (sieving or sieve or comminution or swallow* or saliva or gum or jell* or wax or food)
9. or/5-8

10. TX ("Trail Making Test")
11. (MH "Psychological Tests") OR (MH ”Neuropsychological Tests”)
12. (MH "Health Status Indicators")
13. (MH "Severity of Illness Indices")
14. (MH "Karnofsky Performance Status")
15. (MH "Sickness Impact Profile")
16. (MH "Psychometrics")
17. (MH "Reproducibility of Results")
18. TX (measur* or assess* or evaluat* or test* or inventor* or battery or indicator* or profile or index or indices or scale* or instrument* psychometric* or reproducibility or reliable or reliability or valid*)
19. or/10-18

20. 4 AND 9 AND 19
