# Supplementary material for: Methods for objectively assessing clinical masticatory performance: protocol for a systematic review
Source: Syst Rev. 2017 Jan 26;6:20. doi: 10.1186/s13643-016-0403-5 (PMC5267407; doi:10.1186/s13643-016-0403-5)
Supplement: Additional file 3: — Data extraction form. (DOCX 17 kb) [file 13643_2016_403_MOESM3_ESM.docx]

Methods to objectively assess clinical masticatory performance: Protocol for a systematic review

Additional File 3

Data Extraction Fields for Methods (DRAFT)

| Method ID |  |
| --- | --- |
| Name of method |  |
| Name of person extracting data |  |
| First author |  |
| Year of publication |  |
| Title of article |  |
| Journal |  |
| Country where the method was developed |  |
| Who developed the method |  |
| Purpose of the method |  |
| Study design |  |
| Study sample - Target population for which the method was designed (gender, age) |  |
| Versions of the method |  |
| Version in language other than English |  |
| Who complete the assessment? |  |
| Number of items or stages in the method |  |
| Sections (topics) in the method |  |
| Number of items not assessing masticatory performance |  |
| How is the method scored |  |
| Strengths of the method |  |
| Weaknesses of the method |  |
| Are Adverse events reported – which types and frequencies |  |
| Date of completion of data extraction |  |
| Notes |  |

**Data Extraction Fields for Validation Studies (DRAFT)**

| Name of person extracting data |  |
| --- | --- |
| Method ID |  |
| First author |  |
| Year of publication |  |
| Title of article |  |
| Journal |  |
| Name of journal (if applicable) |  |
| Geographic location of development/validation |  |
| Study design |  |
| Purpose of the paper/study (include hypotheses being tested) |  |
| Study design |  |
| How was the sample obtained? |  |
| Description of the sample (gender, age) |  |
| Were patients asked to provide input on content/face validity? |  |
| Which version of the method is being developed/validated |  |
| Underlying constructs of the method |  |
| Reported time to complete the assessment using the method |  |
| Are Adverse events reported – which types and frequencies |  |
| Readability - scale used and result |  |
| Strengths of the study |  |
| Weaknesses of the study |  |
| Internal structure (factor analysis) |  |
| Response processes |  |
| Standard error of measurement (SEM) |  |
| Correlations with other variables |  |
| Clinically important difference |  |
| Item response theory (IRT)-based analyses |  |
| Includes discussion of internal consistency (section from COSMIN) |  |
| Includes discussion of reliability (section from COSMIN) |  |
| Includes discussion of measurement error (section from COSMIN) |  |
| Includes discussion of content and face validity (section from COSMIN) |  |
| Includes discussion of structural validity (section from COSMIN) |  |
| Includes discussion of hypotheses testing (section from COSMIN) |  |
| Includes discussion of cross-cultural validity (section from COSMIN) |  |
| Includes discussion of criterion validity (section from COSMIN) |  |
| Includes discussion of responsiveness (section from COSMIN) |  |
| Includes discussion of interpretability (section from COSMIN) |  |
| Date of completion of data extraction |  |
| Notes |  |
