# Supplementary material for: Methods for objectively assessing clinical masticatory performance: protocol for a systematic review
Source: Syst Rev. 2017 Jan 26;6:20. doi: 10.1186/s13643-016-0403-5 (PMC5267407; doi:10.1186/s13643-016-0403-5)
Supplement: Additional file 4: — Definitions of measurement properties. (DOCX 16 kb) [file 13643_2016_403_MOESM4_ESM.docx]

Additional file 4. Quality criteria for rating the results of measurement properties

| **Property** | | **Rating** | **Quality criteria** |
| --- | --- | --- | --- |
| Reliability |  |  |  |
|  | Internal consistency | + | Cronbach’s alpha(s) ≥0.70 |
|  |  | ? | Cronbach’s alpha not determined |
|  |  | - | Cronbach’s alpha(s) <0.70 |
|  | Reliability | + | ICC/weighted kappa ≥0.70 or Pearson’s *r*≥0.80 |
|  |  | ? | Neither ICC/weighted kappa or Pearson’s *r* determined |
|  |  | - | ICC/weighted kappa <0.70 or Pearson’s *r*<0.80 |
|  | Measurement errors | + | MIC < SDC or MIC outside the LOA |
|  |  |  | MIC not defined |
|  |  |  | MIC ≤SDC or MIC equals or inside LOA |
| Valdidity |  |  |  |
|  | Content validity | + | The target population considers all items in the questionnaire to be relevant or considers the questionnaire to be complete |
|  |  | ? | No target population involvement |
|  |  | - | The target population considers all items in the questionnaire to be irrelevant or considers the questionnaire to be incomplete |
|  | Structural validity | + | Factors should explain at least 50% of the variance |
|  |  | ? | Factors variance not mentioned |
|  |  | - | Factors explain < 50% of the variance |
|  | Construct validity – hypothesis testing | + | Correlation with an instrument measuring the same construct ≥0.50 or at least 75% of the results are in accordance with the hypotheses and correlation with related constructs is higher than with unrelated constructs |
|  |  | ? | Solely correlations determined with unrelated constructs |
|  |  | - | Correlation with an instrument measuring the same construct <0.50 or <75% of the results are in accordance with the hypotheses or correlation with related constructs is lower than with unrelated constructs |
|  | Cross-cultural validity | + | Original factor structure confirmed or no important DIF between language versions |
|  |  | ? | Confirmatory factor analysis not applied and DIF not assessed |
|  |  | - | Original factor structure not confirmed or important DIF found between language versions |
|  | Criterion validity | + | Convincing arguments that gold standard is “gold” and correlation with gold standard ≥0.70 |
|  |  | ? | No convincing arguments that gold standard is “gold” or doubtful design or method |
|  |  | - | Correlation with gold standard <0.70, despite adequate design and method |
| Responsiveness |  |  |  |
|  | Responsiveness | + | Correlation with an instrument measuring the same construct ≥0.50 or at least 75% of the results are in accordance with the hypotheses or AUC AND correlation with related constructs is higher than with unrelated constructs |
|  |  | ? | Solely correlations determined with unrelated constructs |
|  |  | - | Correlation with an instrument measuring the same construct <0.50 or <75% of the results are in accordance with the hypotheses or AUC OR correlation with related constructs is lower than with unrelated constructs |

SDC, smallest detetable change; LoA, limits of agreement; DIF, differential item functioning; +, positive rating; ?, indeterminate rating; negative rating

Adapted from Terwee et al, J Clin Epidemiol 2007;60(1):34-42, and Dobson et al, Ostheoarthritis and Cartilage, 2012, 20:1548-1562
