# Supplementary material for: Methods for objectively assessing clinical masticatory performance: protocol for a systematic review
Source: Syst Rev. 2017 Jan 26;6:20. doi: 10.1186/s13643-016-0403-5 (PMC5267407; doi:10.1186/s13643-016-0403-5)
Supplement: Additional file 5: — COSMIN checklist domains. (DOCX 14 kb) [file 13643_2016_403_MOESM5_ESM.docx]

Table 5. Definitions of domains, measurement properties, and aspects of measurement properties

| Domain | Measurement property | Aspect of a measurement property | Definition |
| --- | --- | --- | --- |
| Reliability |  |  | The degree to which the measurement is free from measurement error |
| Reliability (extended definition) |  |  | The extent to which scores for patients who have not changed are the same for repeated measurement under several conditions; for example, using different sets of items from the same method (internal consistency) over time (test-retest) by different persons on the same occasion (inter-rater) or by the same person (that is, rates or responders) on different occasions (intra-rater) |
|  | Internal consistency |  | The degree of interrelatedness among the items |
|  | Reliability |  | The proportion of total variance in the measurements which is because of true differences among patients |
|  | Measurement error |  | The systematic and random error of a patient’s score that is not attributed to true change of the construct to be measured |
| Validity |  |  | The degree to which a method measures the construct it purports to measure |
|  | Content validity |  | The degree to which the content of a method is an adequate reflection of the construct to be measured |
|  |  | Face validity | The degree to which the items of a method indeed looks as though they are an adequate reflection of the construct to be measured |
|  | Construct validity |  | The degree to which the scores of a method are consistent with hypotheses(for instance with regard to internal relationships, relationships to scores of other methods, or differences between groups) based on the assumption that the method validly measures the construct to be measured |
|  |  | Structural validity | The degree to which the scores of a method are an adequate reflection of the dimensionalty of the construct to be measured |
|  |  | Hypothesis testing | Idem construct validity |
|  |  | Cross-cultural validity | The degree to which the performance of the items on a translated or culturally adapted method is an adequate reflection of the performance in the items of the original version of the method |
| Responsiveness |  |  | The ability of a method to detect change ovet time in the construct to be measured |
|  | Responsiveness |  | Idem responsiveness |
| Interpretability^a^ |  |  | The degree to which one can assign qualitative meaning, that is, clinical or commonly understood connotations to a method’s quantitative scores or changes in score |

^a^Interpretability is not considered a measurement property but an important characteristic of a measurement method

Adapted from Mokkink LB et al 2010 and Apfelbacher et al 2015
