# Supplementary material for: Methods for objectively assessing clinical masticatory performance: protocol for a systematic review
Source: Syst Rev. 2017 Jan 26;6:20. doi: 10.1186/s13643-016-0403-5 (PMC5267407; doi:10.1186/s13643-016-0403-5)
Supplement: Additional file 6: — Levels of evidence for the quality of the measurement property. (DOCX 13 kb) [file 13643_2016_403_MOESM6_ESM.docx]

Table 6. Levels of evidence for the quality of the measurement property

| Level | Rating* | Criteria |
| --- | --- | --- |
| Strong | +++ or --- | Consistent findings in multiple studies of good methodological quality OR in one study of excellent methodological quality |
| Moderate | ++ or -- | Consistent findings in multiple studies of fair methodological quality OR in one study of good methodological quality |
| Limited | + or - | One study of fair methodological quality |
| Conflicting | ± | Conflicting findings |
| Unknown | ? | Only studies of poor methodological quality |

Adapted from Terwee et al, 2007 and Dobson et al 2012.

* +=positive rating; ?=indeterminate rating; -=negative rating
